# Editorial: Psychological factors in physical education and sport, volume V

**DOI:** 10.3389/fpsyg.2025.1734941

**Published:** 2025-11-24

**Authors:** Giuseppe Battaglia, David Manzano-Sánchez, Carla Chicau Borrego, Manuel Gómez-López

**Affiliations:** 1 Sport and Exercise Research Unit, Department of Psychology, Educational Sciences and Human Movement, University of Palermo, Palermo, Italy; 2 Department of Artistic, Musical and Physical Expression, Faculty of Education, University of Murcia, Murcia, Spain; 3 Sport Sciences School of Rio Maior, Polytechnic Institute of Santarém, SPRINT - Sport Physical Activity and Health Research & Innovation Center, Rio Maior, Portugal; 4 Department of Physical Activity and Sport, Faculty of Sports Sciences, University of Murcia, Murcia, Spain

**Keywords:** motivation, education, sport, physical education, psychological wellbeing

This fifth volume of the “*Psychological Factors in Physical Education and Sport*” series deepens further our understanding of how psychological processes shape learning, performance, and wellbeing across diverse educational and sports settings. This Research Topic includes 37 original and review articles from diverse cultural and methodological perspectives, expanding our understanding of how motivation, cognition, emotion, and social dynamics operate within the context of physical education and sport. The contributions highlight the multidimensional nature of psychological factors related to physical activity and can be grouped into five broad thematic areas: (a) Motivation, autonomy, and psychological needs; (b) Teaching and learning models in physical education; (c) Psychological wellbeing, health, and emotional intelligence; (d) Group dynamics, cohesion, and the social environment; (e) Inclusion and special populations in physical education.

## Motivation, autonomy, and psychological needs

Several studies in this volume focus on motivational processes and the importance of supporting autonomy in sport and educational contexts. Jiang, Razak et al. investigated the relationship between need-supportive coaching behaviors and athletes' engagement, highlighting the mediating role of subjective task value and task orientation. Similarly, Zhang et al. analyzed how a coaching style based on autonomy support fosters resilience and optimism, promoting the overall development of young athletes. Similarly, Langøy et al. showed that when teachers support students' autonomy during physical education classes, it promotes their commitment and the satisfaction of psychological needs, with some possible gender differences. Frikha investigated the relationship between extracurricular physical activity and academic performance, showing that this relationship is mediated by intrinsic motivation and enjoyment. An original study by Valero-Valenzuela et al. demonstrated that a play-based approach (PBA) can enhance motivation and enjoyment among young track and field athletes, suggesting that a PBA program may have positive effects at both the psychosocial and cognitive levels. Similarly, Guo et al. analyzed the relationship between compulsory exercise and motor habits among university students, showing the mediating effects of self-efficacy and attitudes toward exercise. A study by Volgemute et al. revealed that athletes with higher levels of imagery ability achieved better outcomes, suggesting that personalized imagery-based training could improve students' performance. Similarly, Hu, Peng et al. showed the importance of psychological resilience and exercise motivation in promoting sports participation among adolescents. Finally, a scoping review by Haugan et al. examined the relationship between executive functions and “game intelligence” in elite football players, emphasizing the importance of cognitive processes in sports decision-making.

## Teaching and learning models in physical education

A second group of studies examined teaching processes, the role of the teacher, and the educational environment. Su and Liu showed that support from physical education teachers increases students' participation in sports through the mediating effects of learning motivation and self-efficacy. In the same area, Hao et al. found that support from different sources, such as teachers, parents, and peers, positively influences students' motivation to learn in physical education. Wu et al. investigated perceptions of the sports environment and found that they are positively correlated with athletic progress, mediated by self-efficacy and motor behavior. Similarly, Niznikowski et al. explored the effectiveness of verbal feedback in learning complex gymnastics skills, concluding that targeted feedback on key elements significantly improves performance compared to constant feedback on all errors. Finally, Sindiani and Tsuk analyzed the motivations and concerns of motor science students regarding a career as physical education teachers. Their findings highlight the importance of institutional and economic support in influencing students' motivation to pursue a teaching career.

## Psychological wellbeing, health, and emotional intelligence

This area includes several studies that highlight the psychological, emotional, and social benefits of physical activity. Shan et al. showed that participation in soccer can reduce Internet dependence among adolescents, due to the pleasure-mediating role of sports activity. Zheng et al. found that participation in school football improves psychological qualities such as self-esteem, confidence, and sportsmanship. Jiang and Meng investigated the relationship between physical activity and the tendency toward eating disorders among college students, highlighting the protective role of trait positivity and physical self-esteem. Ou et al. explored the association between physical exercise and suicidal ideation, showing that the satisfaction of basic psychological needs reduces the risk of suicidal ideation among university students. A review by Mei et al. and a study by Jiang, Wang et al. confirmed the positive correlation between physical activity and resilience. Furthermore, a meta-analysis by Qiu et al reinforced this evidence, demonstrating that physical activity has a beneficial effect on psychological resilience among young students. From this perspective, Chen et al. explored the relationship between ruminative dispositions, coping strategies, and sports performance in elite athletes, highlighting that rumination can lead to either positive or negative outcomes depending on its focus and the type of coping strategy used. Peng et al. showed that physical exercise reduces feelings of inferiority among college students through the mediating effects of social support and emotional regulation. Similarly, Han et al. demonstrated that physical education is a crucial factor influencing the psychological health of university students. Galán-Arroyo et al. showed the moderating role of perceived physical fitness, suggesting that targeted interventions could help promote emotional wellbeing. A study by Yu et al. identified a positive correlation between university teachers' health, growth mindset, and their self-assessed health status. Zhu et al. showed that exercise combined with additional visual tasks can enhance self-esteem and visual acuity in children. Finally, Meng and Dong indicated that factors such as gender, age, and educational level play a crucial role in shaping attitudes and emotional responses toward physical exercise.

## Group dynamics, cohesion, and the social environment

The fourth group of studies explored interpersonal relationships and the social contexts of sport. Bao showed that peer relationships significantly influence college students' self-efficacy in exercise. The importance of interpersonal relationships, social support, and exercise motivation among university students was further confirmed by the study conducted by Wang et al. Pan and Sui analyzed the coach–athlete relationship, highlighting its positive influence on team performance. In parallel, Zeng and Yin demonstrated that abusive coaching behavior negatively affects athlete engagement. Song et al. investigated the impact of parental psychological control on adolescents' physical activity, identifying a significant negative correlation. Similarly, Cheng and Jiao showed that a positive sports atmosphere enhances subjective wellbeing through peer relationships and positive emotions. Finally, Hu, Zhou et al. proposed a theoretical model based on grounded theory to describe the factors influencing physical activity, identifying individual, family, school, community, and policy-level determinants.

## Inclusion and special populations in physical education

Two articles addressed the topic of inclusion and personal development. Al Harthy et al. examined the relationship between social inclusion and mental health in people with disabilities. Their findings indicated that social inclusion is associated with better overall mental health and a lower incidence of anxiety and depression among individuals with disabilities who participate in sports. Hong et al. described the “Voices of Athletes” program, highlighting its educational benefits and its potential to promote awareness, leadership, and personal development in young athletes.

Based on these concepts, this Research Topic aimed to explore key issues and summarize recent studies on the factors that influence physical and psychological wellbeing, as well as participation in physical activity, in physical education and sport contexts.

